# Thermodynamic analysis of cooperative ligand binding by the ATP-binding DNA aptamer indicates a population-shift binding mechanism

**DOI:** 10.1038/s41598-020-76002-8

**Published:** 2020-11-03

**Authors:** Sladjana Slavkovic, Yanrui Zhu, Zachary R. Churcher, Aron A. Shoara, Anne E. Johnson, Philip E. Johnson

**Affiliations:** 1 Department of Chemistry and Centre for Research on Biomolecular Interactions, York University, Toronto, ON M3J 1P3 Canada; 2 Department of Chemistry and Biology, Ryerson University, Toronto, ON M5B 2K3 Canada

**Keywords:** Biophysical chemistry, DNA, Bioanalytical chemistry

## Abstract

The ATP-binding DNA aptamer is often used as a model system for developing new aptamer-based biosensor methods. This aptamer follows a structure-switching binding mechanism and is unusual in that it binds two copies of its ligand. We have used isothermal titration calorimetry methods to study the binding of ATP, ADP, AMP and adenosine to the ATP-binding aptamer. Using both individual and global fitting methods, we show that this aptamer follows a positive cooperative binding mechanism. We have determined the binding affinity and thermodynamics for both ligand-binding sites. By separating the ligand-binding sites by an additional four base pairs, we engineered a variant of this aptamer that binds two adenosine ligands in an independent manner. Together with NMR and thermal stability experiments, these data indicate that the ATP-binding DNA aptamer follows a population-shift binding mechanism that is the source of the positive binding cooperativity by the aptamer.

## Introduction

Isothermal titration calorimetry (ITC) is a powerful tool for determining the affinity and the thermodynamic parameters of ligand binding from a single experiment^1–3^. ITC is often readily applied in studies of systems where a particular molecule has a single binding site and only one molecule of ligand is bound. However, ITC can also be employed to look at more complex cases where multiple ligands bind at multiple sites on a receptor molecule in either an independent or cooperative manner^4–6^. Previously, we used ITC methods to study two-site binding of cocaine, quinine and quinine-based antimalarial compounds by the cocaine-binding aptamer^7–9^. Aptamers are oligonucleotide molecules that recognize and bind a particular target, which can be a small molecule or ion, a protein or whole cell.

**Figure 1:**
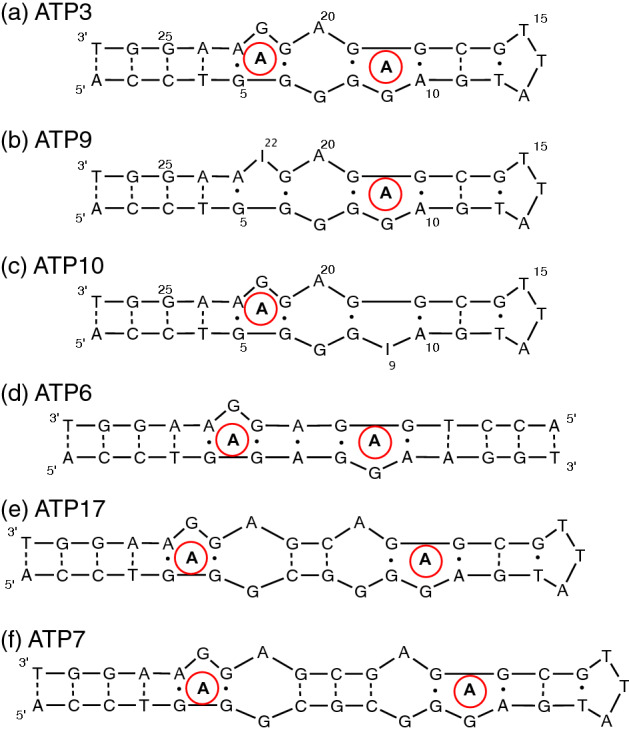
Secondary structures of the ATP-binding aptamers used in this study. (**a**) ATP3, the original 27-nt ATP-binding aptamer; (**b**,**c**) ATP-binding aptamer variants that have inosine substitution at position 22 (ATP9) and position 9 (ATP10); (**d**) The self-complementary 14-nt bimolecular duplex aptamer (ATP6); (**e**,**f**) ATP-binding aptamers with the two binding sites separated by an extra three (ATP17) and four (ATP7) base pairs.

One of the first selected and most frequently studied aptamers is the 27-nucleotide ATP-binding DNA aptamer (ATP3; Fig. 1) that was originally selected by Huizenga and Szostak^10^ and also independently selected by Li and coworkers^11^. This aptamer binds ATP and other adenine-based ligands with micromolar affinity, but it does not bind other nucleotides^10^. The ATP-binding aptamer has been widely used as a model system in many studies looking at biosensor development and employing a wide range of sensing techniques as well as being subject to functional studies^12–22^. The ATP-binding aptamer is also one of the few small molecule-binding aptamers that has had its structure determined. Lin and Patel determined the structure of the ATP-binding DNA aptamer bound to two molecules of AMP using nuclear magnetic resonance (NMR) spectroscopy methods^23^. The structure shows two binding pockets with each pocket occupied by one molecule of AMP. The binding site is in a zipped up internal loop formed by a sheared G·A, and a reverse Hoogsteen G·G mismatch with the unpaired G at the binding site pairing with the AMP ligand (ATP3; Fig. 1).

Despite its widespread use in many ligand binding and biosensor studies, few reports have mentioned the two-site ligand-binding property of the aptamer. As part of a larger study on the ATP-binding aptamer, a recent report included ITC data looking at adenosine binding by the 27-nt aptamer^24^ (ATP3; Fig. 1). The authors reported cooperative binding by the ATP-binding aptamer but were not able to distinguish different affinities at the two ligand-binding sites. Here, we also use ITC methods and have obtained ITC data at six different concentrations from 10 to 100 µM under conditions where the data are clearly non-sigmoidal, an indication of multi-site ligand binding with the binding sites having different affinities. The ITC data were fit individually and using global fitting methods to show that the ATP-binding aptamer follows a cooperative binding mechanism and to determine the binding thermodynamics for both ligand-binding sites.

Binding cooperativity has long been recognized and studied in proteins but has been little studied for aptamer-ligand interactions. Among riboswitches, there is at least one example, the tetrahydrofolate (THF) riboswitch, which has been shown to bind two copies of its ligand cooperatively^25^. Among laboratory-selected aptamers, the ATP binding aptamer was shown to bind its ligand cooperatively. Perhaps because of their rarity when selected, there have been a number of studies to develop cooperative binding aptamers for biotechnology uses. One example is the cooperative binding split aptamer where two cocaine aptamers were fused together and shown to bind two cocaine molecules with positive cooperativity^26^. Additionally, Plaxco and co-workers have developed a method of connecting aptamers with a linker region in order to introduce a population-shift binding mechanism that results in cooperative binding^27–29^. Here, the unbound aptamer is disordered, or poorly ordered, and binding of the first ligand molecule uses some of the total binding free energy (ΔG_bind_) to fold or stabilise the molecule allowing subsequent binding events to have a tighter affinity than they would otherwise have. In this study of the ATP aptamer we combine thermal stability studies, NMR and ITC methods to study the 27-nt classic ATP DNA aptamer as well as two constructs where the binding sites are further apart to propose that this aptamer follows a population shift mechanism which gives rise to its observed positive binding cooperativity.

## Results

### Ligand binding by the ATP-binding aptamer

**Figure 2:**
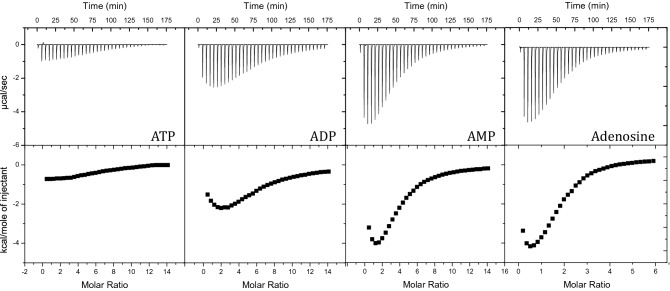
ITC thermograms showing the binding of ATP, ADP, AMP and adenosine by the aptamer ATP3. (Top) The raw titration data showing the heat resulting from each injection of adenine-type ligand into aptamer solution. (Bottom) The integrated heat plot after correcting for the heat of dilution. Data acquired at 20 °C in 10 mM sodium acetate buffer (pH 5.5), 120 mM NaCl and 70 µM ATP3.

We used the originally selected 27-nucleotide ATP-binding aptamer^10^ (ATP3; Fig. 1) to look at binding by the aptamer to a variety of adenine-type ligands. We observed binding by the ATP3 aptamer to ATP, ADP, AMP and adenosine (Fig. 2, Supplementary Figure S1). The ITC data for these ligands were fit to both a cooperative and an independent binding model. The model that provided the lowest residual sum of squared differences (RSS) between the experimental and calculated data points was judged as the best fit (Table 1). Thus, from the analysis of the data in Table 1, at 70 µM DNA, the cooperative binding model resulted in the best fit for all the adenine-type ligands. We then chose adenosine for more detailed analysis, as it provided the best quality ITC data in terms of signal-to-noise ratio.

**Table 1:** Affinity and thermodynamic binding parameters for the binding of the different ligands by the ATP-binding aptamer (ATP3) used in this study. Data are fit to two different binding models.

Model	Ligand	K_d1_ (µM)	ΔH_1_ (kcal mol^−1^)	ΔS_1_ (kcal mol^−1^)	K_d2_ (µM)	ΔH_2_ (kcal mol^−1^)	ΔS_2_ (kcal mol^−1^)	RSS^a^	n_H_
Cooperative	ATP	133 ± 55	− 1.8 ± 0.1	− 3.4 ± 0.3	498 ± 123	− 6.0 ± 1.7	1.5 ± 1.6	9.7 × 10^6^	1.2
Independent		1.9 ± 1.9	− 0.83 ± 0.02	− 6.8 ± 0.6	186 ± 28	− 1.3 ± 0.1	− 3.7 ± 0.1	2.9 × 10^11^	–
Cooperative	ADP	101 ± 33	− 2.7 ± 0.1	− 2.7 ± 0.2	254 ± 37	− 13 ± 1	8 ± 1	1.0 × 10^7^	1.2
Independent		1.3 ± 0.8	− 1.5 ± 0.1	− 6.4 ± 2.6	87 ± 10	− 2.8 ± 0.2	− 2.6 ± 0.1	2.2 × 10^11^	–
Cooperative	AMP	86 ± 26	− 6.7 ± 0.1	1.2 ± 0.1	127 ± 9	− 16 ± 1	15 ± 1	1.3 × 10^7^	1.3
Independent		0.8 ± 0.4	− 3.7 ± 0.1	− 4.5 ± 0.3	89 ± 3	− 6.7 ± 0.2	0.9 ± 0.4	4.4 × 10^11^	–
Cooperative	Adenosine	42 ± 13	− 11 ± 3	4.9 ± 3.2	43 ± 2	− 27 ± 2	21 ± 2	5.6 × 10^7^	1.3
Independent		0.7 ± 0.3	− 4.5 ± 1.2	− 3.7 ± 1.3	24 ± 1	− 10 ± 1	4.1 ± 0.2	2.9 × 10^11^	–

Data acquired at 20 °C in 10 mM sodium acetate (pH 5.5), 120 mM NaCl at 70 µM DNA.

^a^RSS is the residual sum of squared differences between experimental and calculated data points.

**Figure 3:**
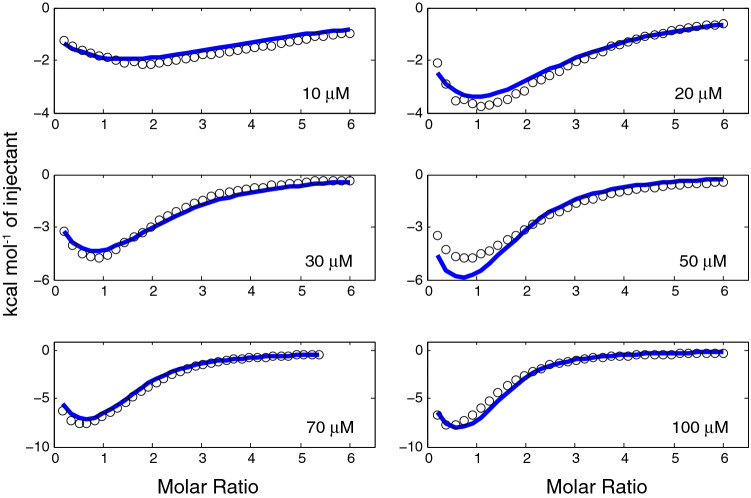
Global fit to the cooperative binding model showing the binding of adenosine to ATP3 acquired at six aptamer concentrations. Shown in open black circles are the experimental ITC data points and the blue solid line shows the global fit. Data acquired at 20 °C in 10 mM sodium acetate buffer (pH 5.5), 120 mM NaCl.

In order to confirm that ATP3 binds two molecules of adenosine in a cooperative and not an independent manner, we acquired ITC binding data at 6 different aptamer concentrations, from 10 to 100 µM. These data were fit to the cooperative and independent binding models both individually and globally in a manner previously used by us^7^ as described by Freiburger et al.^5^. For each individual data set at each different aptamer concentration, the model providing the lowest RSS value was judged as the best fit (Table 2). For all six aptamer concentrations, the cooperative model provided the best fit of the experimental data to the calculated ones. The ITC data from the experiments performed at the six different concentrations were also analysed using a global fit to both the independent and cooperative binding models (Supplementary Table S1, Fig. 3 and Supplementary Figure S2). Also in this case, the cooperative model provided the best fit as judged by the lowest RSS values and from a visual inspection of Fig. 3 and Supplementary Figure S2.

**Table 2:** Affinity and thermodynamic parameters for adenosine binding by ATP3 at the different aptamer concentrations used in this study fit to the independent and cooperative binding models.

Model	[Aptamer] (µM)	K_d1_ (µM)	ΔH_1_ (kcal mol^−1^)	ΔS_1_ (kcal mol^−1^)	K_d2_ (µM)	ΔH_2_ (kcal mol^−1^)	ΔS_2_ (kcal mol^−1^)	RSS^a^
Cooperative	10	6.1 ± 1.4	− 1.7 ± 0.1	− 5.3 ± 0.2	50 ± 33	− 17 ± 11	11 ± 12	1.3 × 10^9^
Independent		1.5 ± 2.5	− 0.2 ± 0.6	− 7.5 ± 1.1	21 ± 32	− 4.7 ± 4.4	− 2.1 ± 4.5	1.3 × 10^12^
Cooperative	20	17 ± 9	− 3.5 ± 0.2	− 2.9 ± 0.3	32 ± 11	− 24 ± 6	19 ± 6	9.8 × 10^8^
Independent		0.6 ± 0.5	0.3 ± 17	− 8.7 ± 17	12 ± 1	− 5.2 ± 0.6	− 1.4 ± 0.6	1.6 × 10^12^
Cooperative	30	15 ± 2	− 4.1 ± 0.7	− 2.4 ± 0.1	26 ± 2	− 21 ± 1	15 ± 1	1.8 × 10^8^
Independent		0.7 ± 0.3	− 1.9 ± 0.4	− 6.4 ± 0.5	15 ± 1	− 7.4 ± 0.4	0.9 ± 0.4	3.4 × 10^11^
Cooperative	50	26 ± 3	− 5.2 ± 0.6	− 0.9 ± 0.6	57 ± 18	− 23 ± 2	18 ± 2	1.6 × 10^7^
Independent		1.9 ± 0.9	− 1.9 ± 2.7	− 5.8 ± 2.8	35 ± 1	− 8.2 ± 0.7	2.2 ± 0.2	2.2 × 10^11^
Cooperative	70	42 ± 13	− 11 ± 3	4.9 ± 3.2	43 ± 2	− 27 ± 2	21 ± 2	5.6 × 10^7^
Independent		0.7 ± 0.3	− 4.5 ± 1.2	− 3.7 ± 1.3	24 ± 1	− 10 ± 1	4.1 ± 0.2	2.9 × 10^11^
Cooperative	100	45 ± 4	− 11 ± 2	4.8 ± 1.5	50 ± 7	− 26 ± 2	20 ± 2	6.0 × 10^7^
Independent		0.6 ± 0.2	− 4.4 ± 1.0	3.9 ± 1.0	35 ± 1	− 8.7 ± 0.1	2.7 ± 0.1	1.9 × 10^12^
Cooperative fit average		25 ± 15	− 6 ± 3	− 0.3 ± 0.4	43 ± 12	− 23 ± 4	17 ± 4	2.6 × 10^9^
Independent fit average		1.0 ± 1.0	− 2.1 ± 2.1	− 6 ± 2	24 ± 9	− 8 ± 3	1.1 ± 2.4	4.2 × 10^12^

Data acquired at 20 °C in 10 mM sodium acetate buffer (pH 5.5), 120 mM NaCl.

^a^RSS is the residual sum of squared differences between experimental and calculated data points.

The thermodynamic parameters for adenosine binding at each aptamer concentration as well as the averages of the individual fits are shown in Table 2. The thermodynamic binding parameters from the global fit are shown in Supplementary Table S1. There is agreement between the average of the individual fits and the global fit to the cooperative model within the reported error range for all values except for the − TΔS_1_ values (Table 2, Supplementary Table S1).

### NMR analysis of adenosine binding

**Figure 4:**
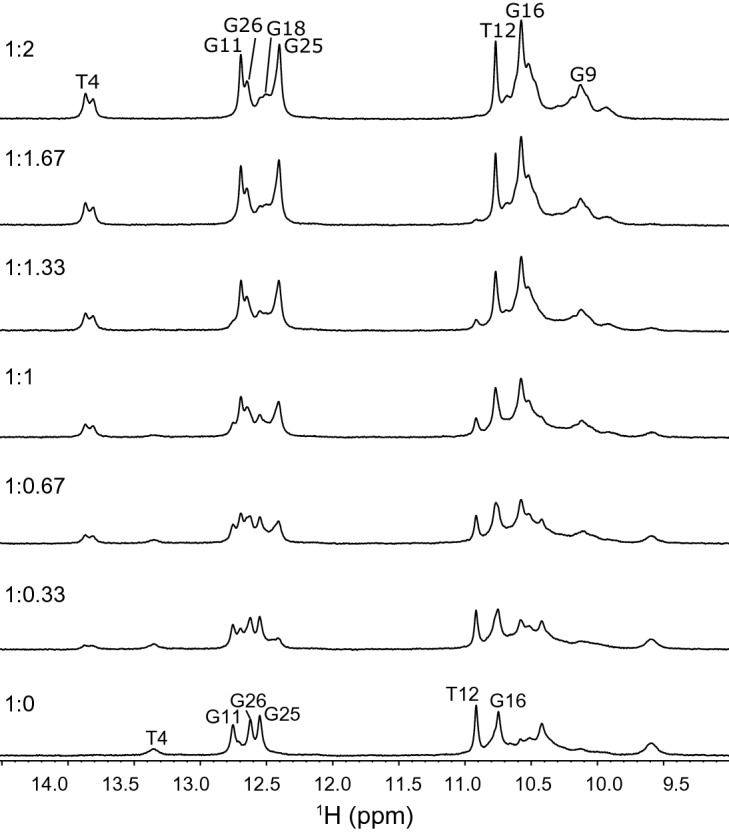
Adenosine binding monitored by one-dimensional (1D) ^1^H NMR. Displayed is the region of the NMR spectrum focused on the imino resonances as a function of increasing adenosine concentration. All spectra were acquired at 5 °C in 10 mM ammonium acetate-d_7_ buffer (pH 5.5), 120 mM NaCl in 10% ^2^H_2_O/90% ^1^H_2_O.

To gain some insight into the structure of the ATP3 aptamer and how adenosine binding affects the aptamer structure we titrated adenosine into a solution of ATP3 while monitoring the one-dimensional ^1^H NMR spectra (Fig. 4). Additionally, we performed 2D NOESY experiments on both the free and adenosine-bound ATP3 to obtain assignments of as many imino protons as possible (Supplementary Figure S3). The NMR spectra of free ATP3 were quite poor due to a high degree of line broadening and signal overlap. For the adenosine-bound ATP3, the data were of better quality but there is still a high degree of signal overlap and weak cross peaks in the NOESY between non-Watson–Crick base pairs that allowed only a limited subset of imino protons to be assigned. The assignments we made are consistent with those reported previously for ATP3 bound to AMP and are consistent with ATP3 forming a hairpin structure in both free and bound forms as reported by Lin and Patel^23^. Binding of adenosine to ATP3 is in slow exchange on the NMR timescale.

### Binding site mutations

In order to see how the two binding sites in the ATP-binding aptamer are affected by a mutation that eliminates binding at one site, but not the other, we introduced a guanine to inosine change at position 9 (ATP10; Fig. 1) and position 22 (ATP9; Fig. 1). A previous study by Lin and Patel demonstrated that the guanine to inosine substitution at these positions resulted in no binding^23^. The ITC thermograms for ATP9 and ATP10 are shown in Supplementary Figure S4 with both showing only very weak binding. These data were fit to a one-site binding model with affinities for adenosine of (212 ± 69) µM for ATP9 and (188 ± 37) µM for ATP10.

### One-site ATP-binding aptamers

In order to test the importance of the base pairs immediately adjacent to the binding site we analysed the one-site ATP-binding aptamer Apt1d originally published by Liu and co-workers^24,30^ as well as two related aptamers Apt1d-GC1 and Apt1d-GC2 (Supplementary Figure S5). In Apt1d-GC1 the G·G mismatch next to the G that interacts with the ligand was changed to be a G–C base pair. For Apt1d-GC2 both the G·G mismatch and the G·A mismatch next to the G7 binding site were changed to be G–C base pairs. As seen in Supplementary Figure S5 the Apt1d aptamer binds adenosine while neither Apt1d-GC1 nor Apt1d-GC2 shows any indication of binding adenosine.

### Binding by a bimolecular duplex ATP-binding aptamer

The ATP-binding aptamer can also be made as a bimolecular duplex DNA using a 14-nucleotide self-complementary strand^31^. This aptamer, ATP6 (Fig. 1), binds adenosine (Supplementary Figure S5) and best fits the cooperative binding model on the basis of the calculated RSS values (Supplementary Table S2).

### Design of an ATP-binding aptamer with two independent binding sites

In order to provide insight into the role played by proximity of the two ligand-binding sites, we designed two ATP-binding aptamers with binding sites separated by different distances, as measured by base pairs, compared to the ATP3 aptamer (Fig. 1). These constructs are ATP17 and ATP7, which contained three and four additional base pairs, respectively. The binding of both ATP17 and ATP7 to adenosine was analysed using ITC methods (Supplementary Figures S7, S8) with binding by ATP17 being best fit with a 2-site cooperative model and the binding by ATP7 being best fit by a two-site independent model as judged by the RSS values (Table 3).

**Table 3:** Binding thermodynamics, affinities and Hill coefficient (n_H_) for adenosine binding by the ATP aptamers of different length.

Model	Aptamer	K_d1_ (µM)	ΔH_1_ (kcal mol^−1^)	ΔS_1_ (kcal mol^−1^)	K_d2_ (µM)	ΔH_2_ (kcal mol^−1^)	ΔS_2_ (kcal mol^−1^)	RSS^a^	n_H_
Cooperative	ATP3	45 ± 4	− 11 ± 2	4.8 ± 1.5	50 ± 7	− 26 ± 2	20 ± 2	6.0 × 10^7^	1.3
Independent		0.6 ± 0.2	− 4.4 ± 1.0	3.9 ± 1.0	35 ± 1	− 8.7 ± 0.1	2.7 ± 0.1	1.9 × 10^12^	–
Cooperative	ATP17	44 ± 8	− 1.0 ± 0.1	− 4.7 ± 0.1	129 ± 71	− 12 ± 2	7 ± 2	3.7 × 10^7^	1.1
Independent		12 ± 2	0.1 ± 0.5	− 7 ± 1	624 ± 206	− 1.7 ± 0.3	− 3.4 ± 0.6	1.3 × 10^10^	–
Cooperative	ATP7	0.6 ± 0.3	− 7.7 ± 0.1	− 1.1 ± 0.2	48 ± 6	− 11 ± 2	4 ± 2	4.4 × 10^11^	–
Independent		1.3 ± 0.5	− 6.4 ± 0.1	− 2.0 ± 0.6	32 ± 1	− 12 ± 1	4 ± 1	9.3 × 10^10^	–

Data acquired at 20 °C in 10 mM sodium acetate (pH 5.5), 120 mM NaCl at a 100 µM DNA aptamer concentration.

^a^RSS is the residual sum of squared differences between experimental and calculated data points.

### Stability of ATP-binding aptamers

**Figure 5:**
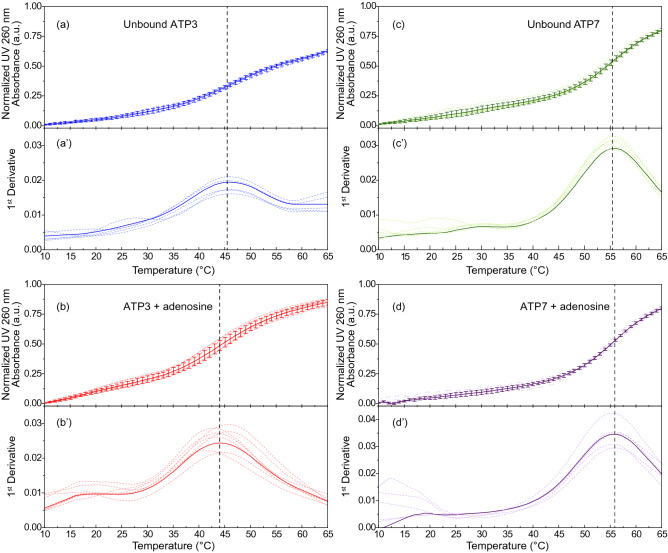
Thermal stability analysis using UV melting curves. Shown is the normalized UV absorbance at 260 nm in a temperature range of 10 °C to 65 °C for (**a**,**a**′) the unbound ATP3 aptamer (**b**,**b**′) ATP3 bound to adenosine (**c**,**c**′) the unbound ATP7 aptamer and (**d**,**d**′) ATP7 bound to adenosine. The prime symbols indicate the first derivative analyses of the corresponding melting. Faint dotted curves represent acquired normalized data for each replicate, and solid lines denote the average of each experiment with error bars for the standard deviations in 5–6 replicates. Dashed lines designate the *T*_*m*_ points of unbound aptamer and adenosine-bound complexes. Data acquired in 10 mM sodium acetate buffer (pH 5.5) and 120 mM NaCl.

To assess the effect on the aptamer stability of introducing base pairs between the two binding sites in ATP3, we used temperature-scanning UV experiments to measure the melting temperatures of ATP3 and ATP7 both free and bound to adenosine. For ATP3, the melting temperature is (45.2 ± 0.5) °C for the free aptamer and (44.2 ± 1.5) °C for the adenosine-bound form. For ATP7, the free aptamer melts at (55.6 ± 0.5) °C while adenosine-bound ATP7 unfolds at (55.8 ± 0.6) °C (Fig. 5). For both aptamers, this demonstrates that ligand binding does not appreciably influence the melting temperature and that ATP7 has a significantly higher melting temperature than ATP3.

## Discussion

In this work, we used ITC methods to determine that the ATP-binding aptamer binds two copies of its ligand in a positive cooperative manner. The well-established 27-nt ATP aptamer sequence (Fig. 1; ATP3) binds ATP, ADP and AMP as well as adenosine. This binding of a range of adenine-based ligands is consistent with what has been reported earlier^10^. The ITC data for all ligands studied best fit a cooperative model as opposed to a two-site independent model (Table 1). The two ligand-binding sites in the ATP3 aptamer are very similar as the nucleotides in the immediate vicinity of the binding sites are the same, but the two sites are not identical (Fig. 1). One binding site is closer to the loop than the other site and the G·A mismatch between the two ligand-binding sites is not symmetrical. The shape of the ITC thermograms we acquired (Fig. 2) indicates there are two binding sites with different affinities. The non-equivalency of the two sites was confirmed by the data best fitting a cooperative binding model with the two sites having different affinities (Table 1). In a previous ITC study, the affinity of the two ligand-binding sites were not distinguishable which may be related to the different buffer used and the higher aptamer concentration employed in our study^24^. We do note that we determined the Hill coefficient of ATP3 for adenosine to be 1.3. This closely matches the n_H_ value of 1.2 determined in the previous study^24^.

From our binding results of the ATP-binding aptamer with the different ligands (Table 1) it is clear that as the number of phosphate groups on the ligand is reduced, the affinity at both binding sites becomes stronger. This is particularly notable in the weaker binding site where the K_d2_ values change from (498 ± 123) µM for ATP to (43 ± 2) for adenosine (Table 1). In the structure of the ATP3 aptamer, the two binding sites are adjacent on the same side of the helical structure^23^. We believe that binding became tighter with fewer phosphate groups on the ligands due to electrostatic repulsion between the two negatively charged ligands as well as the negatively charged DNA. In support of this, the neutral ligand adenosine is the tightest binding molecule (Table 1).

Adenosine was chosen for an in-depth study as it provided the best quality data, being the most exothermic ligand and having the tightest binding affinity among the different ligands studied (Table 1). In order to further confirm that binding follows the cooperative model, ITC data were acquired at six different ATP3 aptamer concentrations. These data best fit the cooperative model when analysed either globally or individually (Fig. 3, Supplementary Figure S2; Table 2, Supplementary Table S1). In support of the data fitting results, with the exception of the − TΔS_1_ value, the thermodynamic parameters of binding match within the error range when comparing the average values of the individual fits (Table 2) with the results of the global fit (Supplementary Table S1). The binding affinities we report are slightly weaker than values previously reported that were not able to distinguish between the two binding sites and range from (6 ± 3) to (16.4 ± 1.4) µM^10,24^. Aside from fitting the data to different binding models, this difference could result from different experimental buffer conditions such as temperature, salt concentration, pH or concentration of DNA used.

In order to test if cooperativity is observed in different constructs of the ATP DNA aptamer, binding by the bimolecular duplex version of the aptamer was tested using ITC methods. The bimolecular duplex version of the ATP-binding aptamer (ATP6; Fig. 1) was originally studied by Lin and Patel as it provided improved ^1^H-NMR spectra compared to ATP3^31^. The binding of adenosine by ATP6 also best fits the cooperative binding model, with a Hill coefficient value of 1.4, though this aptamer binds adenosine weaker than ATP3 (Supplementary Table S2). The exact reason why weaker binding is observed remains unknown though it likely arises, at least partly, from having an A·A mismatch between the binding sites instead of the G·A mismatch in ATP3. It is possible the three-dimensional structure is altered in a way that hinders binding. Alternately, the unbound ATP6 structure may need more free energy from ligand binding to fold than ATP3 which is a hairpin and contains a G·A mismatch.

The ATP-binding aptamer is a well-cited example of a structure-switching aptamer where the unfolded aptamer is disordered, or has a disordered region, which becomes ordered upon ligand binding^17,23^. This structural transition was demonstrated by Lin and Patel who monitored the structure of the aptamer by NMR spectroscopy where the free aptamer had fewer and broader imino ^1^H signals than seen in the ATP aptamer bound to two molecules of AMP^23^. We also observed this ligand-induced structural ordering, as new peaks in the imino ^1^H region of the NMR spectrum of ATP3 appeared when we added adenosine (Fig. 4). The NMR titration shows a concerted binding process where both binding sites in the aptamer become ordered with ligand binding as opposed to a sequential process where one of the sites binds adenosine and becomes ordered, then the second site binds adenosine and becomes ordered.

We propose a mechanism for the observed positive binding cooperativity that follows the population-shift binding mechanism. The mechanism involves the unbound ATP aptamer having an unstructured region consisting of the two binding sites and the intervening stem region. This structural arrangement is consistent with the existing NMR data. When the first adenosine molecule binds the aptamer, some of the free energy of binding (ΔG_b_) goes into ordering, or reducing the motion or dynamics, at both the first and second binding sites and in the region between the sites. The remaining ΔG_b_ is reflected in the apparent binding affinity of the first ligand. The structural ordering at the second site, resulting from binding at the first, allows the second adenosine ligand to bind tighter than it otherwise would, resulting in the observed positive cooperativity.

To test this proposed mechanism, we determined the binding affinity of two aptamer constructs where we separated the two binding sites by an increased distance by introducing new base pairs between the binding sites. These are the ATP17 and ATP7 constructs. To keep the binding site environment as unchanged as possible, we introduced into these constructs a G·A mismatch as well as G–C base pairs. When introducing these base pairs, the binding sites will get further apart, and it will likely be less able for binding at one site to influence the structure of the other: in other words, negating the population-shift binding model. Also, the overall stability of the molecule should increase. This increase in stability is inevitable as new base pairs are introduced. Indeed, the melting temperature for ATP7 was higher (by ~ 10 °C) than that of ATP3 in both the free and bound aptamer forms (Fig. 5). As the two sites are separated, we propose the intervening region forms a stable stem and the two binding sites become isolated and binding at one site now causes no changes in the structural ordering or dynamics of the other. These isolated sites would then best fit an independent binding model. In this case, the binding of the first ligand should be tighter than in the construct where the sites are closer, and binding is cooperative, as less of the ΔG_b_ needs to be used to alter the structure or dynamics at the second site and in the intervening region and more ΔG_b_ would be observed in the apparent binding affinity. As explained below, this is what we observe.

When comparing ligand binding by ATP3, ATP17 and ATP7 (Table 3) we observe that the degree of cooperativity as reflected in the value of the Hill coefficient, drops from 1.3 in ATP3 to 1.1 for ATP17. The affinity at the weaker site (K_d2_) in the aptamer is reduced in ATP17 (129 ± 71) µM compared to ATP3 (50 ± 7) µM. This is consistent with the two sites becoming less linked, as they are becoming separated and more of the binding free energy at the second site is needed to reduce the dynamics at that site and less ΔG_b_ would be reflected in the measured apparent binding affinity.

For the ATP7 aptamer, adenosine binding is no longer cooperative and the data now best fits a two-site independent model. This is likely due to the combined effect of the two sites being separated and further apart in distance and the structural disorder or dynamics at the two sites no longer being linked. Additionally, the ATP7 aptamer itself would be more stable, and likely less dynamic due to the introduction of the stem between the two sites.

In support of the disorder at the binding site being linked to the binding affinity, we tested two constructs where in each aptamer one of the binding sites was eliminated by changing the guanosine that interacts with the ligand to be an inosine (ATP9, ATP10)^23^. Both ATP9 and ATP10 retain only very weak single-site binding to adenosine (Supplementary Figure S4). In these aptamers, the ligand does not bind at the altered binding site but the unbound aptamer should retain its disordered nature at the entire binding site. With binding at the one remaining site still possible, more of the binding free energy from that single ligand binding event needs to go into folding the aptamer with much weaker binding being observed.

In contrast to the constructs with guanosine to inosine mutations in the binding sites (ATP9, ATP10) a single-sited ATP-binding aptamer has been developed where one of the sites has been removed and replaced with Watson–Crick base pairs (Apt1d; Supplementary Figure S5)^30^. As part of a previous study, we determined the affinity of Apt1d to be (21 ± 2) µM which matches the affinity reported by Zhang and Liu and is much tighter than what we see for ATP9 and ATP10^32^. Additionally, this value is within the error range of the high affinity site in ATP3 (Table 2 and Supplementary Table S1). This comparatively strong affinity reflects the removal of a disordered binding site and replacing it with an ordered region of Watson–Crick base pairs. As a result, the aptamer retains its affinity as now there is no region outside of the immediate binding site that becomes ordered with ligand binding at the cost of some of the binding free energy. We noted that the function of adenosine binding needs the presence of the mismatched G·G and A·G base pairs immediately adjacent to the binding site as the mutations in the constructs Apt1d-GC1 and Apt1d-GC2 eliminated ligand binding (Supplementary Figure S5).

The conclusion from this study is that the ATP-binding aptamer consistently follows a cooperative binding mechanism whether it is the original 27-nt sequence (hairpin duplex) or the bimolecular duplex version and regardless of the ligand the aptamer binds (Table 1). We propose that the positive cooperativity in the ATP aptamer is explained by a population-shift binding mechanism where the two binding sites are linked and the initial binding event affects the structure or dynamics at both binding sites and in the region between the two. Due the structuring of the first binding event, the second ligand therefore binds tighter than it otherwise would.

## Methods

### Materials

Aptamer samples were purchased from Integrated DNA Technologies (IDT, Coralville, Iowa) with standard desalting and used without further purification. DNA samples were dissolved in distilled, deionized water and then exchanged three times in a 3 kDa molecular weight cutoff concentrator with 1 M NaCl and washed at least three times with distilled deionized water. All DNA samples, with the exception of Apt1d, Apt1d-GC1 and Apt1d-GC2, were exchanged in 10 mM sodium acetate buffer (pH 5.5) and 120 mM NaCl three times before use. Apt1d, Apt1d-GC1 and Apt1d-GC2 samples were run in 10 mM HEPES (pH 7.6), 100 mM NaCl, 2 mM MgCl_2_. Aptamer concentrations were determined by ultraviolet absorbance spectroscopy using the calculated extinction coefficients provided by the manufacturer. ATP, ADP, AMP and adenosine were all obtained from Sigma Aldrich. Stock solutions of ligands for binding experiments were prepared by dissolving the appropriate weight of ligand into buffer.

### Nuclear magnetic resonance spectroscopy

NMR experiments were conducted on a 600 MHz Bruker Avance spectrometer equipped with a ^1^H–^13^C–^15^ N triple resonance probe. The ATP3 sample was heated in boiling water for 1 min, and then cooled in an ice-water bath for at least 5 min to favor intramolecular folding of the aptamer prior to performing NMR experiments. All 1D ^1^H spectra were acquired in 10 mM ammonium acetate-d_7_ buffer (pH 5.5), 120 mM NaCl in 10% ^2^H_2_O / 90% ^1^H_2_O at 5 °C with a 1.8 mM ATP3 sample. A two-fold molar excess of adenosine to aptamer was added to ATP3 to obtain the adenosine-bound ATP3 sample. A 2D ^1^H–^1^H NOESY was performed on the free and adenosine-bound ATP3 sample with a mixing time (τ_m_) of 200 ms. Water suppression for all experiments was achieved using excitation sculpting^33^.

### Stability studies by UV melting experiments

UV melting experiments on the aptamers ATP3 and ATP7, both free and adenosine-bound, were performed using a Cary 100 UV–Vis spectrometer and 10-mm fused quartz cuvettes. The rate of temperature increase for each experiment was 1 °C/min as controlled by a Cary Peltier unit with two data points acquired per minute in a temperature range from 8 °C to 83 °C. Data was analysed in a range of 10 °C to 65 °C to eliminate background signals. The different aptamer conditions were performed with 5–6 replicates. Each experiment was performed in 10 mM sodium acetate buffer (pH 5.5) and 120 mM NaCl. For each ligand-aptamer complex, solutions were filter-sterilized using a 0.2 µm microfilter. A concentration of the aptamer was chosen to yield ~ 0.5 absorbance arbitrary units (a.u.) at 260 nm using extinction coefficients of the aptamer. The ligand to aptamer molar ratio was kept constant at 95% ligand-bound using Eq. (1):

1 $$X = \left[ {\text{L}} \right]^{n} /\left( {K_{{\text{d}}}^{n} + \left[ {\text{L}} \right]^{n} } \right)$$

where [L] is the ligand concentration, *X* is the fraction bound, *n* equals 2 binding events and *K*_*d*_ is the dissociation constant at 20 °C as determined in this study. To quantify the thermal shift, the first derivative of each thermal curve was plotted as a function of temperature using OriginPro 2016 software (OriginLab Corporation, Northhampton, MA, USA), as described previously^34^.

### Isothermal titration calorimetry

ITC binding experiments were performed using a MicroCal VP-ITC instrument in a manner similar to what we previously described^35^. Samples were degassed before analysis with a MicroCal Thermo Vac unit. All experiments were corrected for the heat of dilution of the titrant. Titrations were performed with the aptamer samples in the cell and the ligand as the titrant, in the needle. All aptamer samples were heated in a 95 °C water bath for 3 min and cooled in an ice water bath prior to use in a binding experiment to allow the DNA aptamer to anneal in an intramolecular fashion.

The binding experiments were performed at 20 °C with the aptamer solution at a concentration of 10 to 100 µM using adenosine concentrations of 0.312 to 2.8 mM. Apt1d, Apt1d-GC1 and Apt1d-GC2 samples were run at 100 µM DNA at 20 °C. All binding experiments consisted of an initial delay of 60 s, a first injection of 2 µL and then a 300 s delay. Subsequent 34 injections were 8 µL, spaced every 300 s. The first point was removed from all data sets due to the different injection volume and delay parameters.

ITC data was fit to both cooperative and two-independent sites binding models described by Freiburger et al.^5^ using MATLAB 14 software. Data following a one-set of sites model was analyzed using the manufacturer provided Origin 7.0 software. The ITC data at aptamer concentrations of 70 µM for ATP3, ATP6, ATP17 and ATP7 were analysed to determine the Hill coefficient (n_H_) using established methods^5,36,37^.

## Supplementary information

Supplementary Information.
