# Supplementary material for: Thermodynamic analysis of cooperative ligand binding by the ATP-binding DNA aptamer indicates a population-shift binding mechanism
Source: Sci Rep. 2020 Nov 3;10:18944. doi: 10.1038/s41598-020-76002-8 (PMC7609719; doi:10.1038/s41598-020-76002-8)
Supplement: Supplementary file 1 — Supplementary Information. [file 41598_2020_76002_MOESM1_ESM.pdf]

Supplementary Data

Thermodynamic analysis of cooperative ligand binding by the ATP-binding DNA aptamer indicates a population-shift binding mechanism

Sladjana Slavkovic[1], Yanrui Zhu[1], Zachary R. Churcher[1], Aron A. Shoara[1], Anne E. Johnson[2], Philip E. Johnson[1,*]

[1]Department of Chemistry and Centre for Research on Biomolecular Interactions, York University, Toronto, Ontario, Canada, M3J 1P3.

[2]Department of Chemistry and Biology, Ryerson University, Toronto, Ontario, Canada, M5B 2K3.

* To whom correspondence should be addressed: Tel: 416-736-2100 x3319; Email: pjohnson@yorku.ca

Supplementary Table S1. Binding of adenosine by the ATP3 aptamer at six different concentrations using a global fit to the cooperative and independent binding model.[1]

model	K_{d1} (μM)	ΔH_1 (kcal mol^{-1})	$-T\Delta S_1$ (kcal mol^{-1})	K_{d2} (μM)	ΔH_2 (kcal mol^{-1})	$-T\Delta S_2$ (kcal mol^{-1})	RSS[2]
Cooperative	28 ± 5	-7.0 ± 0.3	1.0 ± 0.3	36 ± 4	-22 ± 1	16 ± 1	4.2×10^{7}
Independent	1.2 ± 0.7	4.7 ± 0.2	-12 ± 0.4	45 ± 18	-11 ± 11	5 ± 11	2.2×10^{11}

[1]Data acquired at 20°C in 10 mM sodium acetate buffer (pH 5.5), 120 mM NaCl. [2]RSS is the residual sum of squared differences between experimental and calculated data points.

Supplementary Table S2. Affinity and thermodynamic parameters of binding for the independent and cooperative models for adenosine binding by the ATP6 aptamer.[1]

model	Aptamer	K_{d1} (μM)	ΔH_1 (kcal mol^{-1})	$-T\Delta S_1$ (kcal mol^{-1})	K_{d2} (μM)	ΔH_2 (kcal mol^{-1})	$-T\Delta S_2$ (kcal mol^{-1})	RSS[2]	n_H
Cooperative	ATP6	91 ± 42	-3.0 ± 0.1	-2.9 ± 0.3	279 ± 18	-57 ± 21	-50 ± 21	4.4×10^{8}	1.4
Independent		0.9 ± 0.8	-3.7 ± 0.1	-4.5 ± 0.6	226 ± 27	-9 ± 1	3 ± 1	1.1×10^{12}	-

[1]Data acquired at 20°C in 10 mM sodium acetate buffer (pH 5.5), 120 mM NaCl. [2]RSS is the residual sum of squared differences between experimental and calculated data points.

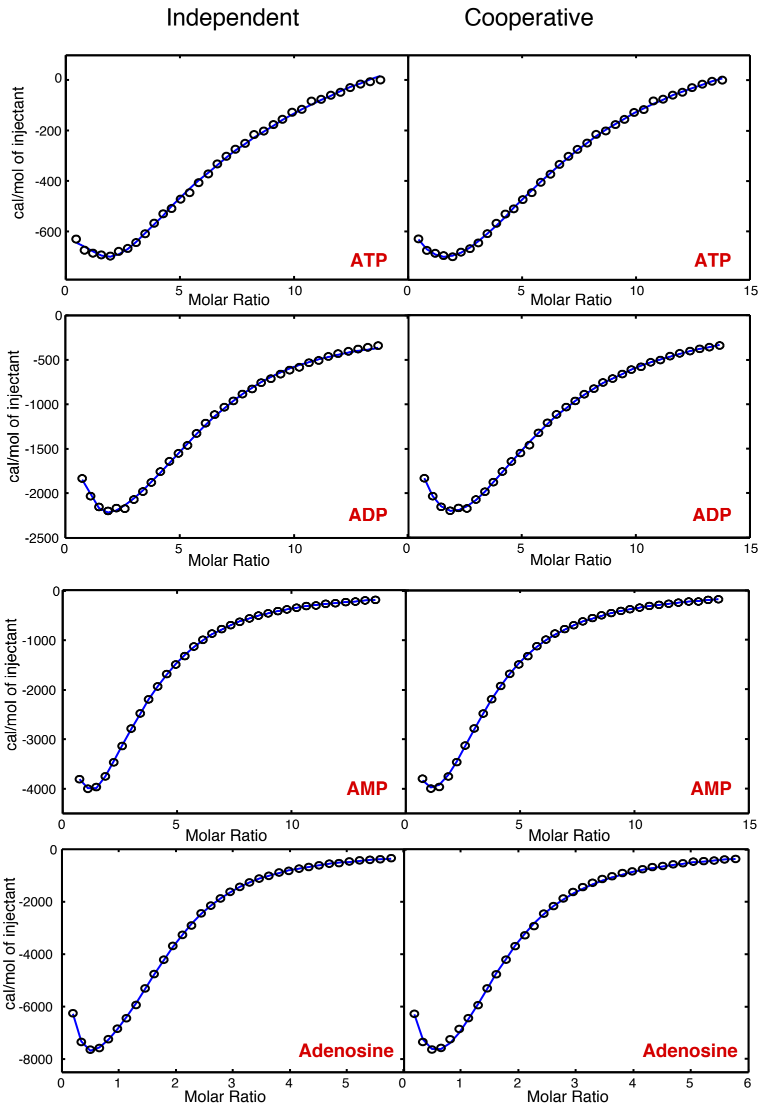

Supplementary Figure S1. ITC data showing the binding of ATP, ADP, AMP and adenosine to the ATP3 aptamer. The binding data for ATP, ADP, AMP and adenosine (open black circles) was fit to both independent and cooperative binding models (blue line). Data were acquired at 20°C in 10 mM sodium acetate buffer (pH 5.5), 120 mM NaCl.

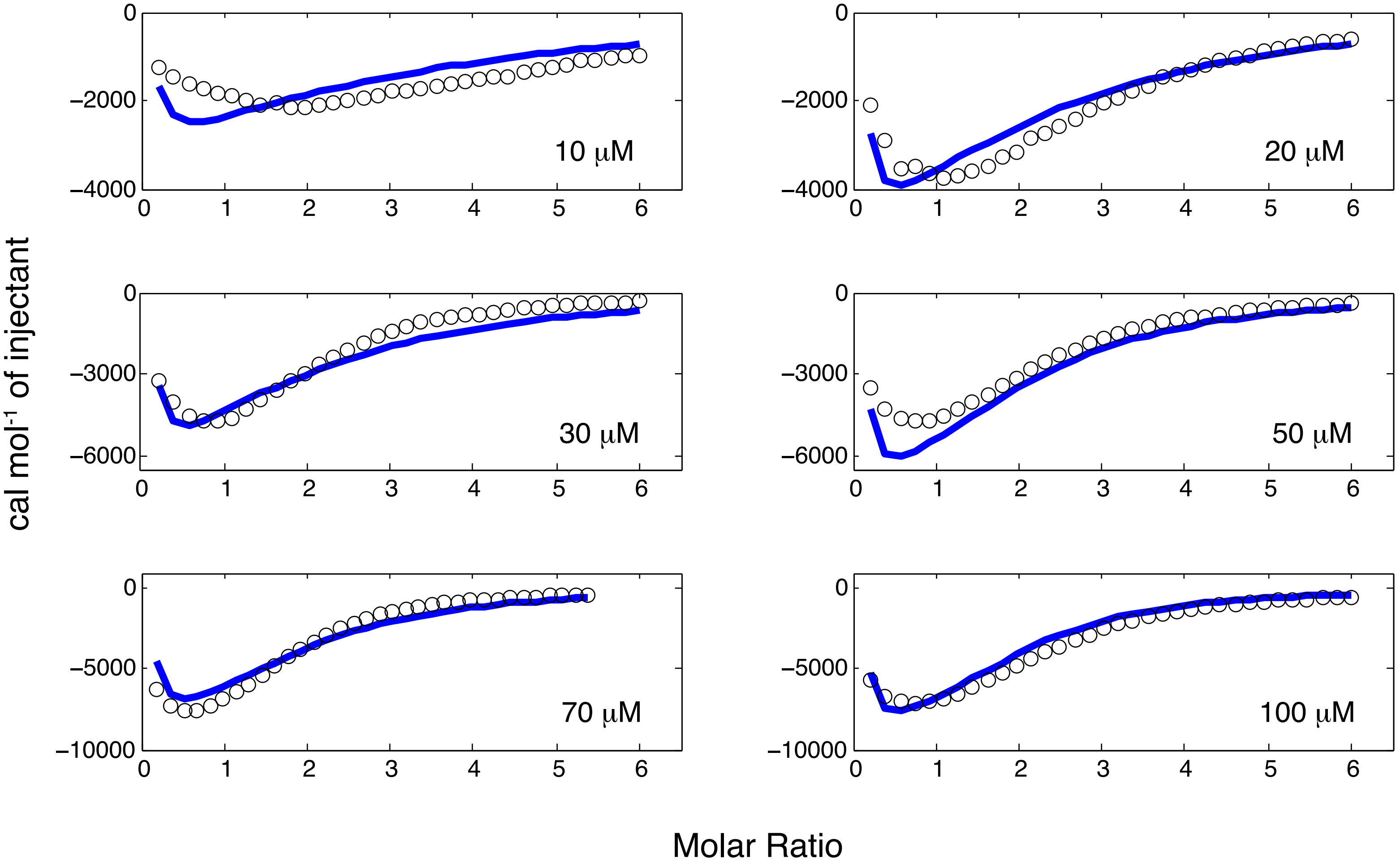

Supplementary Figure S2. Global fit to the independent binding model showing binding of adenosine to ATP3 acquired at six aptamer concentrations. Shown in open black circles are the experimental data points and the blue solid line shows the global fit. Data acquired at 20°C in 10 mM sodium acetate buffer (pH 5.5), 120 mM NaCl.

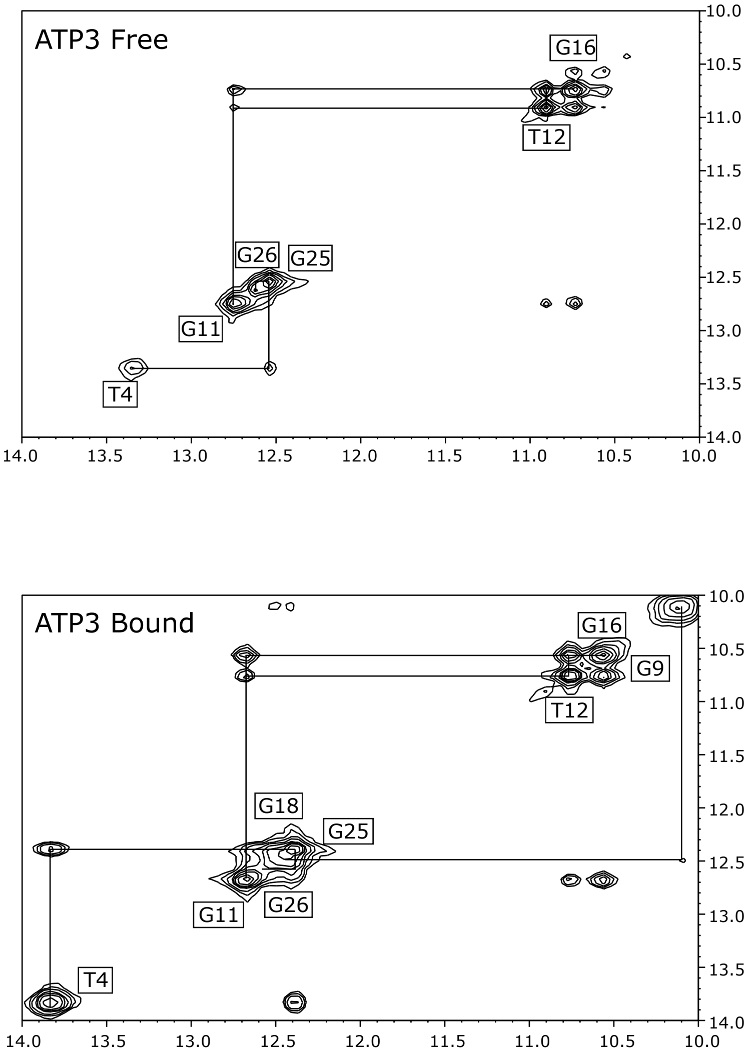

Supplementary Figure S3. 2D NOESY imino-proton assignments of free (top) and adenosine-bound (bottom) ATP3. Data acquired at 5°C in ammonium acetate-d_7 buffer (pH 5.5), 120 mM NaCl in 10% ^2H_2O / 90% ^1H_2O, with a mixing time of 200ms.

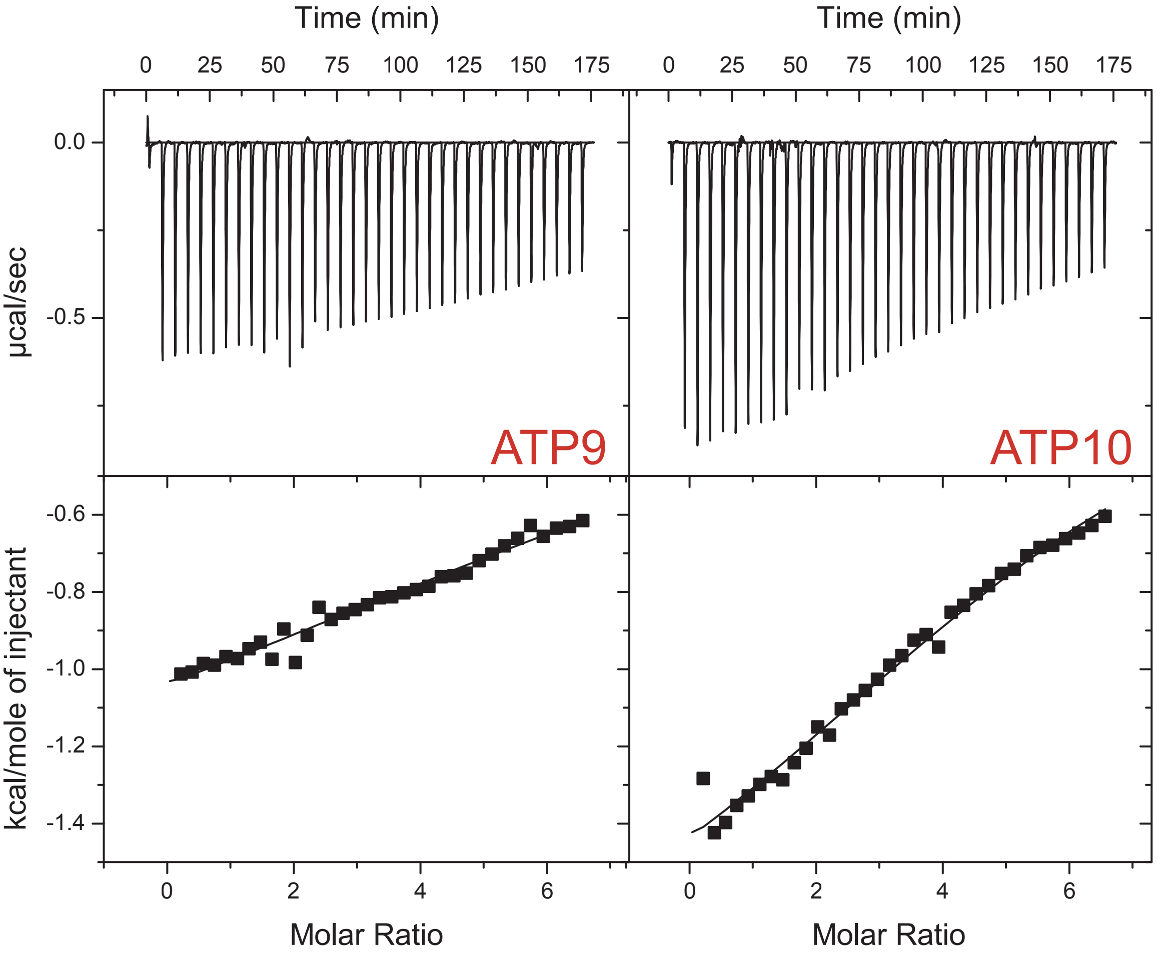

Supplementary Figure S4. ITC thermograms showing binding of adenosine to the ATP9 and ATP10 aptamers. (Top) The raw titration data showing the heat resulting from each injection of adenosine into aptamer solution. (Bottom) The integrated heat plot after correcting for the heat of dilution. Data were fit to a single-site binding model and acquired at 20°C in 10 mM sodium acetate buffer (pH 5.5), 120 mM NaCl.

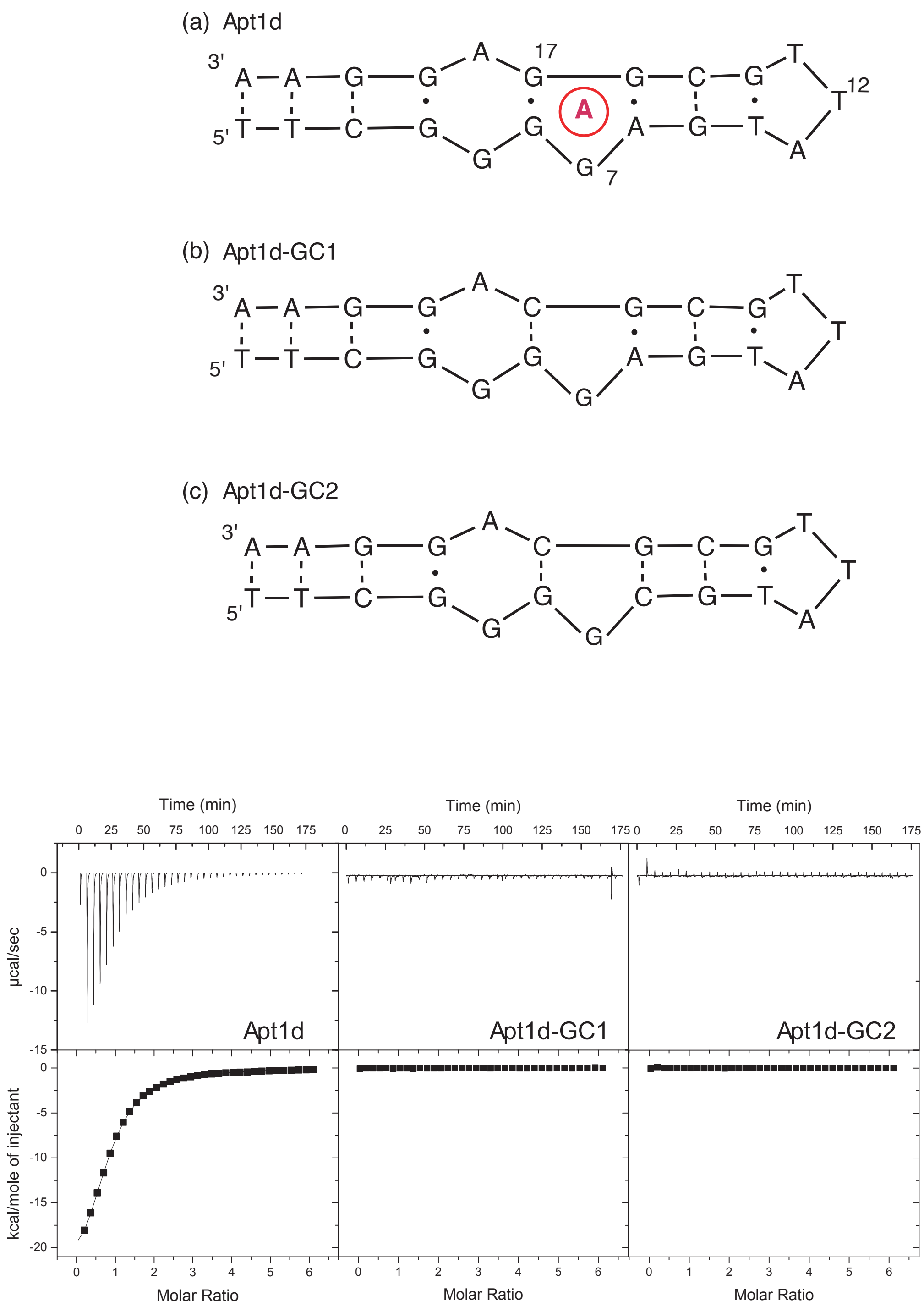

Supplementary Figure S5. (top) Secondary structures of the one-sited ATP-binding aptamers Apt1d, Apt1d-GC1 and Apt1d-GC2. Dashed lines indicate Watson-Crick base pairs while dots indicate non-Watson-Crick base pairs. (bottom) ITC thermograms showing binding of adenosine to Apt1d, Apt1d-GC1 and Apt1d-GC2. Data acquired at aptamer concentrations of 100 µM DNA at 20°C in 10 mM HEPES (pH 7.6), 100 mM NaCl, 2 mM $MgCl_2$.

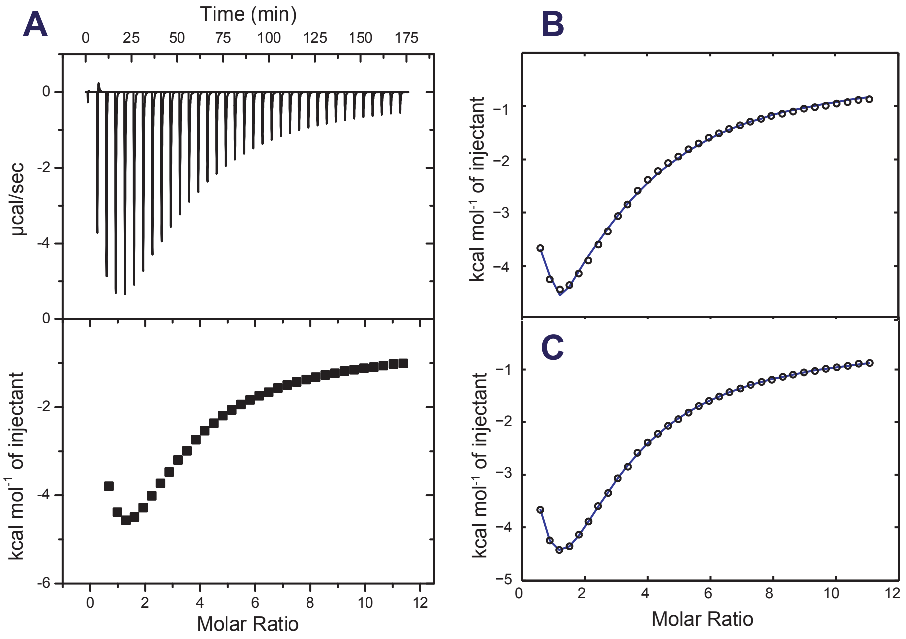

Supplementary Figure S6. (A) ITC data showing the binding of adenosine to the ATP6 aptamer. The binding data were fit to (B) an independent sites and (C) a cooperative sites model. Open black circles represent the experimental data and the blue solid line represents the theoretical fit. Data acquired at 20°C in 10 mM sodium acetate buffer (pH 5.5), 120 mM NaCl.

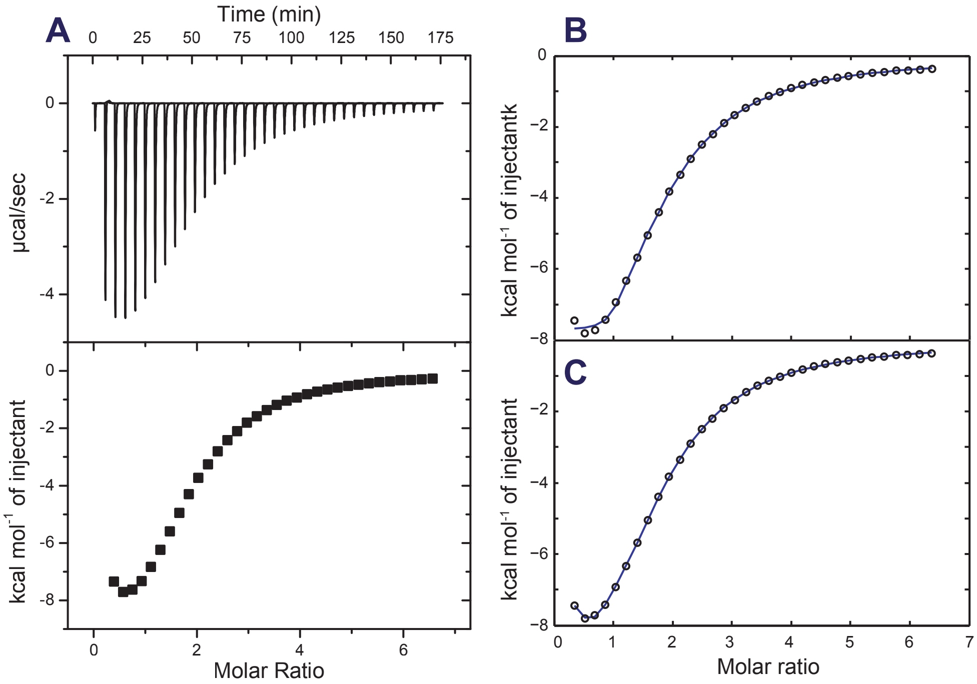

Supplementary Figure S7. (A) ITC data showing the binding of adenosine to the ATP7 aptamer. The binding data were fit to (B) an independent sites and (C) a cooperative sites model. Open black circles represent the experimental data and the blue solid line represents the theoretical fit. Data acquired at 20°C in 10 mM sodium acetate buffer (pH 5.5), 120 mM NaCl.

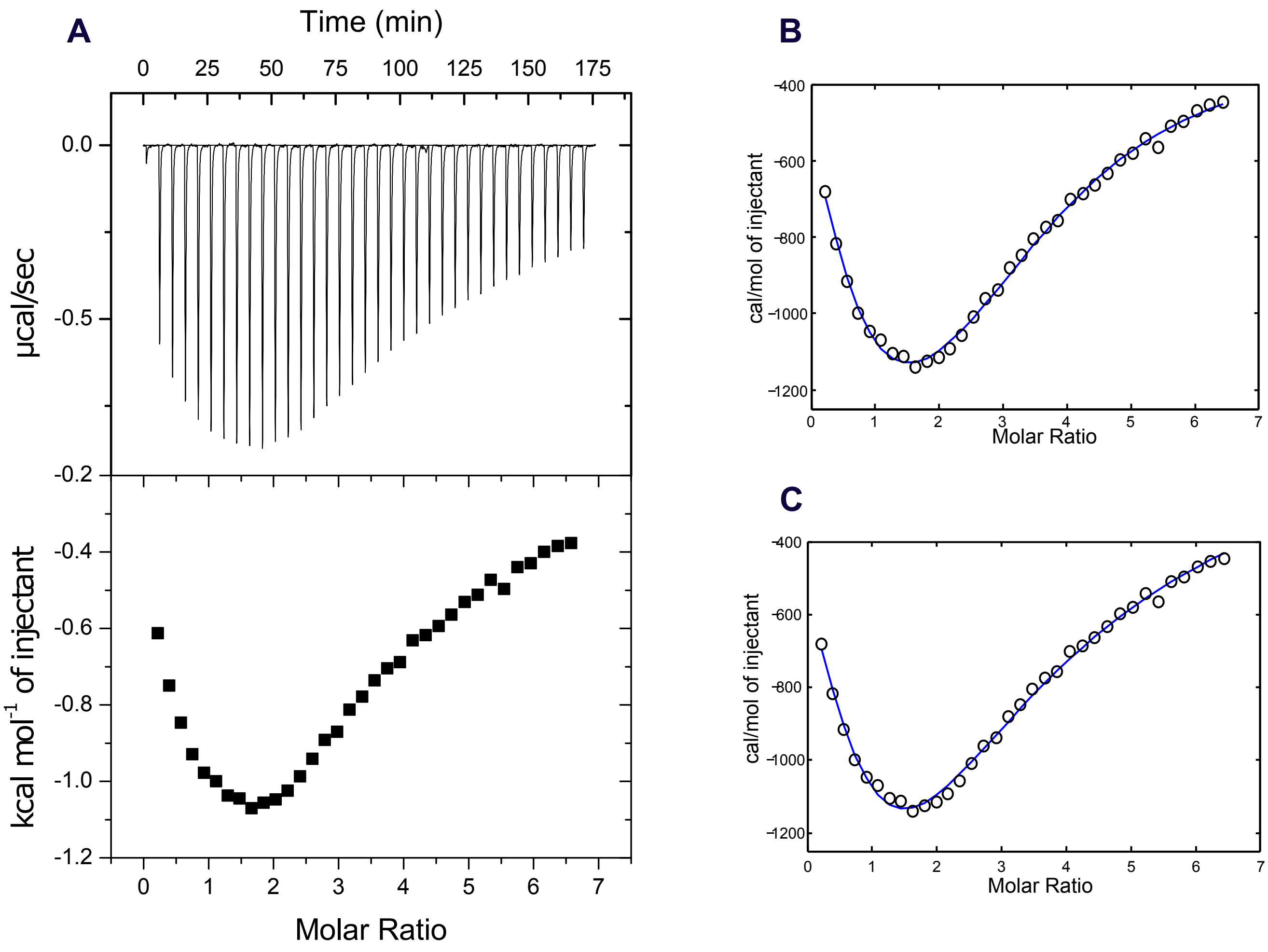

Supplementary Figure S8. (A) ITC data showing the binding of adenosine to the ATP17 aptamer. The binding data was fit to (B) an independent sites and (C) cooperative sites model. Open black circles represent the experimental data and the blue solid line represents the theoretical fit. Data acquired at 20°C in 10 mM sodium acetate buffer (pH 5.5), 120 mM NaCl.
